# Dental Caries

**Published:** 1880-05

**Authors:** 


### TRANSLATION.

### DENTAL CARIES.

In his chirurgical report of the hospital of Liege, Dr. Jansen, regimental physician, publishes in the *Archives medical belges* some considerations on dental caries of a very practical character, which we think fit to reproduce here.

Every day the soldiers suffering from odontalgia are sent to the hospital to have extracted one or more teeth, more or less decayed. Extraction is not always indispensable; cauterisation, filling, and the use of narcotics *inwardly* and *outwardly*, are sometimes very efficacious.

The decay of a tooth may be the origin of the inflammation of the gums and the swelling of the cheek.

In these circumstances, men are sent to the hospital to be treated. We have had in our military service a large number of patients suffering from this affection. Let us say a few words about the caries in question. Evidently there exists an hereditary predisposition to it, but it is especially observed in northern climates and in low and wet countries.

It has been wrongly maintained that it is always observed in pulmonary tuberculosis.

In syphilis the teeth decay to a large extent on account of the malady, and not from the use of mercury.

Dental caries, to be sure, is found in children born with syphilitic affection (Stromeyer). The milk teeth appear more quickly, but they are dark and brittle. The mercurial stomatis often causes falling out of the teeth. The misuse of sugared preparations is especially hurtful to first dentition. Very warm and very cold drinks frequently bring about the destruction of the dental substance. Traumatic lesions of the enamel do not always degenerate into caries, and broken teeth may very well serve the purposes of mastication without becoming painful.

Preventive means ought never to be neglected. The mouth ought to be kept in the greatest state of cleanliness; after meals it must be washed out carefully with water and the teeth cleaned with a soft brush or a bit of sponge. As soon as a decayed place becomes evident it must be removed and fillings applied. If the nerve of the teeth is exposed, it must be first cauterized with phenic acid. As an economical means of filling, gutta-percha is very advantageous.

By odontologia is meant affections of the most varied kind. We have frequently observed, especially in Antwerp and in the forts of the lower Escant, intermittent toothaches of a paludic nature. Sulphate of quinine in these cases is recommended with perfect assurance. In anemia, the result of heavy fevers, these cases are advantageously treated by tonics. Sometimes they are of a rheumatic character, and are then frequently complicated with headache. After having treated then the saburral state, if it exists, extract of aconite is prescribed. When the gums become inflamed and give place to the formation of abscess, the collutoires (gum-wash), emollient and slightly narcotic, quiet the pains and favor the opening of the abscess. Sometimes, however, it is necessary to have recourse to the lancet. Toothache may sometimes depend on chronic osteitis and the jawbone, which necessitates the iodure (iodine without acid) of potassium.

Toothache is caused by cerebral congestion on the consequences of internal and external decay. The afflux or rush of blood toward the head is brought about by heat and the use of strong drinks. They must be combatted by intestinal derivations. As to neuralgia of the teeth, properly so called, it requires the use of morphine, chloral or chloroform.

One must never be in too great a hurry to have decayed teeth extracted, especially when they can be still used for mastication. Recourse ought to be had to this operation when no remedy calms the pain, when filling is not possible, when there exists abundant suppuration, a fistule, (gum-boil), or when the breath is fetid. I recommend patients to content themselves with luxation of the decayed tooth.

In the case of simple caries, this operation may be efficacious, since, or as its effect is to divide the dental nerve ; but, in the case of alveolitis, extraction is indispensable.

Our cases of instruments for dental operations contain two Garengert keys, a pair of straight forceps, a pair of bent forceps, and gouges or elevators. With this small number of instruments a skillful sergeant ought to be able to extract any tooth. Incisors and canine teeth are taken out with the forceps, the molars with the keys and the roots with the gouges or elevators. The key is the most serviceable instrument in dentistry. As early as 1840, Liston declared that the key had been abandoned for the forceps. However, it still continues to be much used, and it would be difficult to find an instrument to take its place. Specialists who use the forceps exclusively ought to have a large number of them. It is well then to wrap the handle with batting, which should be kept in place by a narrow band or string of cotton, in such a manner that the movement of the hook is not impeded. It ought to be placed on the outside part of the tooth, excepting in the case of the last molar. The key may be used for removing roots, but then very small or fine hooks are necessary.

The most efficacious means for stopping the bleeding following the extraction of the tooth, is the pressure or thrusting into the aperture or opening, a piece of raw cotton saturated with perchloride of iron.—*Le Progrès Dentaire.* E. B. M.

